# Supplementary material for: Time-resolved proteomic profiling reveals compositional and functional transitions across the stress granule life cycle
Source: Nat Commun. 2023 Nov 27;14:7782. doi: 10.1038/s41467-023-43470-1 (PMC10682001; doi:10.1038/s41467-023-43470-1)
Supplement: Supplementary file 1 — Supplementary Information [file 41467_2023_43470_MOESM1_ESM.pdf]

Time-resolved proteomic profiling reveals compositional and functional transitions across the stress granule life cycle.

Shuyao Hu *et al*.

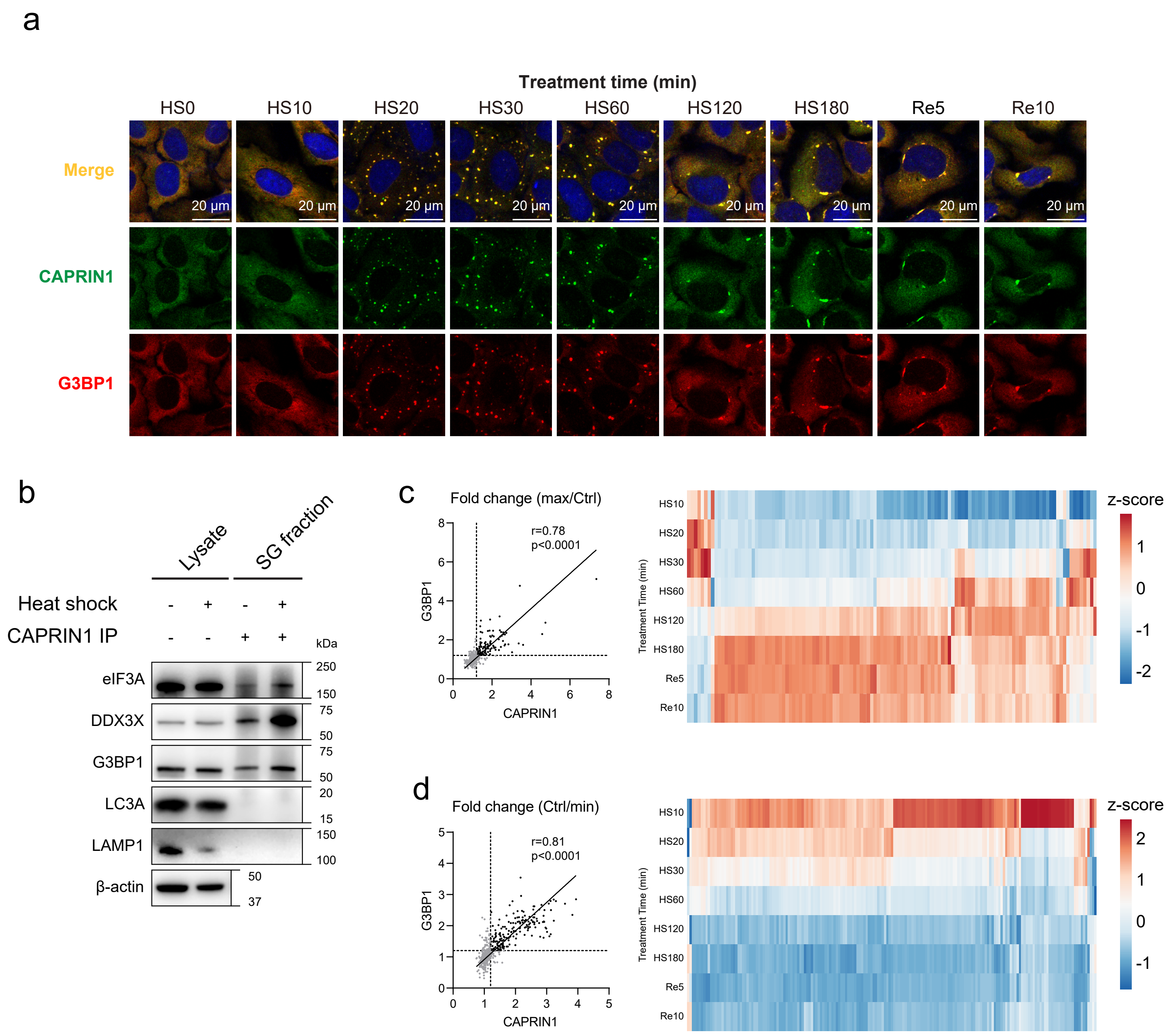

Supplementary Fig. 1. SGs across the heat-shock and recovery cycle, and the identification of SG-enriched proteins, related Figure 1.

a. Confocal micrographs of U2OS cells fixed at the indicated time points during HS and recovery; representative images from 3 biological replicates are shown.

b. Immunoblots of the total input and the SG fraction samples collected under normal growth or heat shock conditions, as indicated; the detection of SG constituents (eIF3A, DDX3X, G3BP1) or non-SG organelles (autophagosome as indicated by LC3A and lysosome as indicated by LAMP1); representative blots from 3 biological replicates are shown.

c-d. Fold change selection of time-resolved proteomic profile and hierarchical clustering heatmap of selected SG proteins. The scatter plot (left panel) compares the max/control (c) and control/min (d) fold change values of the CAPRIN1 dataset and G3BP1 dataset, n = 442 proteins; linear regression line is plotted on each graph; r and P value of Pearson's correlation are labeled on each graph (Pearson's r were determined by comparing CAPRIN1 vs. G3BP1 data set. P values were calculated using two-sided t-test).

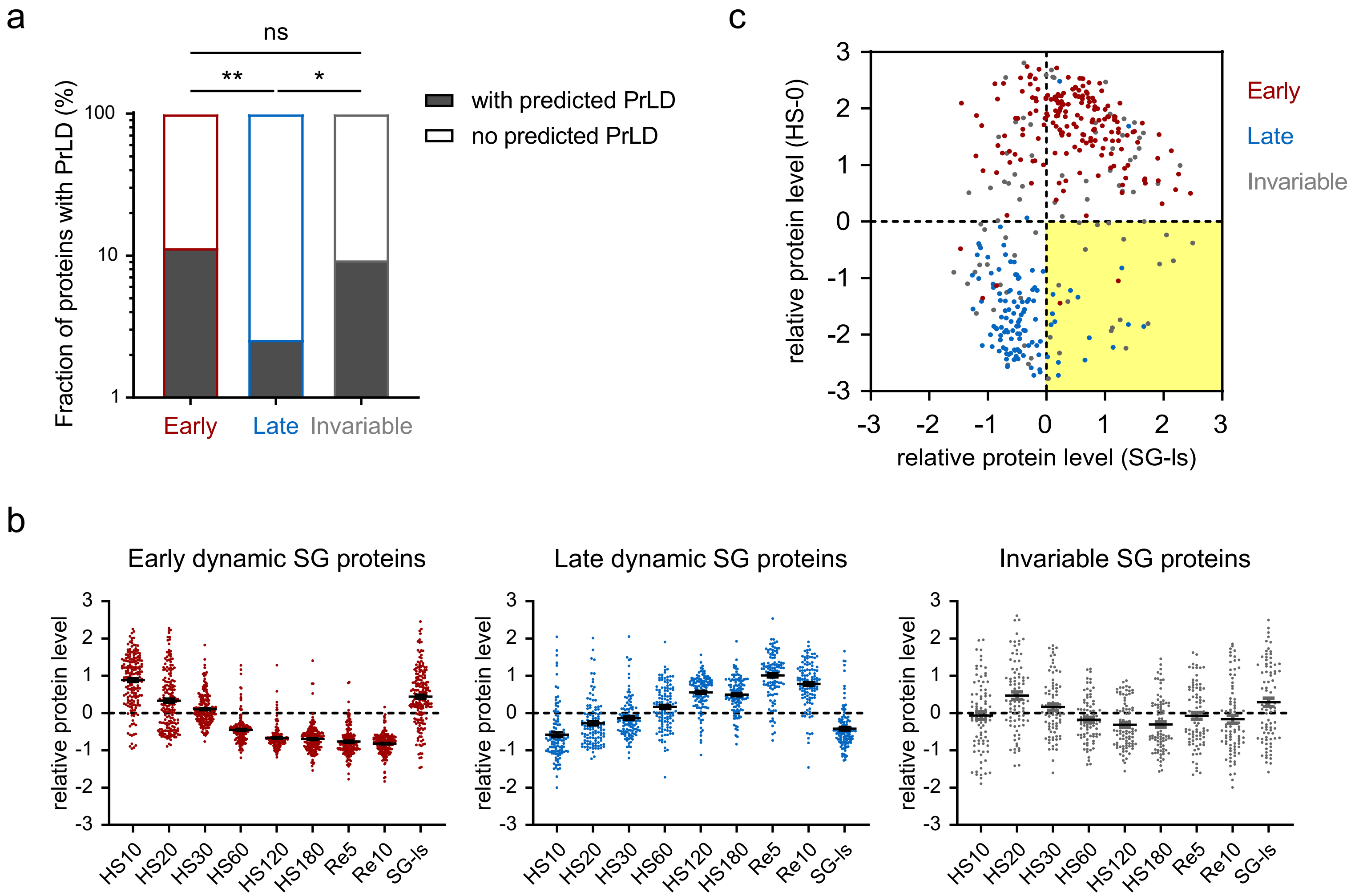

Supplementary Fig. 2. Comparisons among three groups of SG proteins, related to Figure 2.

a. Bar graph showing the percentage of proteins with annotated PrLDs in each SG protein groups, with early dynamic proteins, late dynamic proteins and invariable proteins shown in red, blue, and grey, respectively; *P < 0.05, **P <0.01, ns not significant (two-sided Chi-square test, exact P values: early vs. late, 0.0061; early vs. invariable, 0.6227; late vs. invariable, 0.0356).

b. Relative protein levels under different HS, recovery and control conditions are plotted, with early dynamic proteins, late dynamic proteins and invariable proteins plotted separately in red, blue, and grey, respectively; n = 184, 116, and 85 proteins for early, late and invariable proteins, respectively; error bars indicate SEM.

c. Scatter plot showing relative protein levels, calculated as z-scores, in SG-ls and G3BP1-IP samples; data points for early dynamic proteins, late dynamic proteins and invariable proteins are shown in red, blue, and grey, respectively; the quadrant, where data points represent protein-enrichment in SG-ls but not inHS-0 control, is highlighted in yellow; n = 385 proteins.

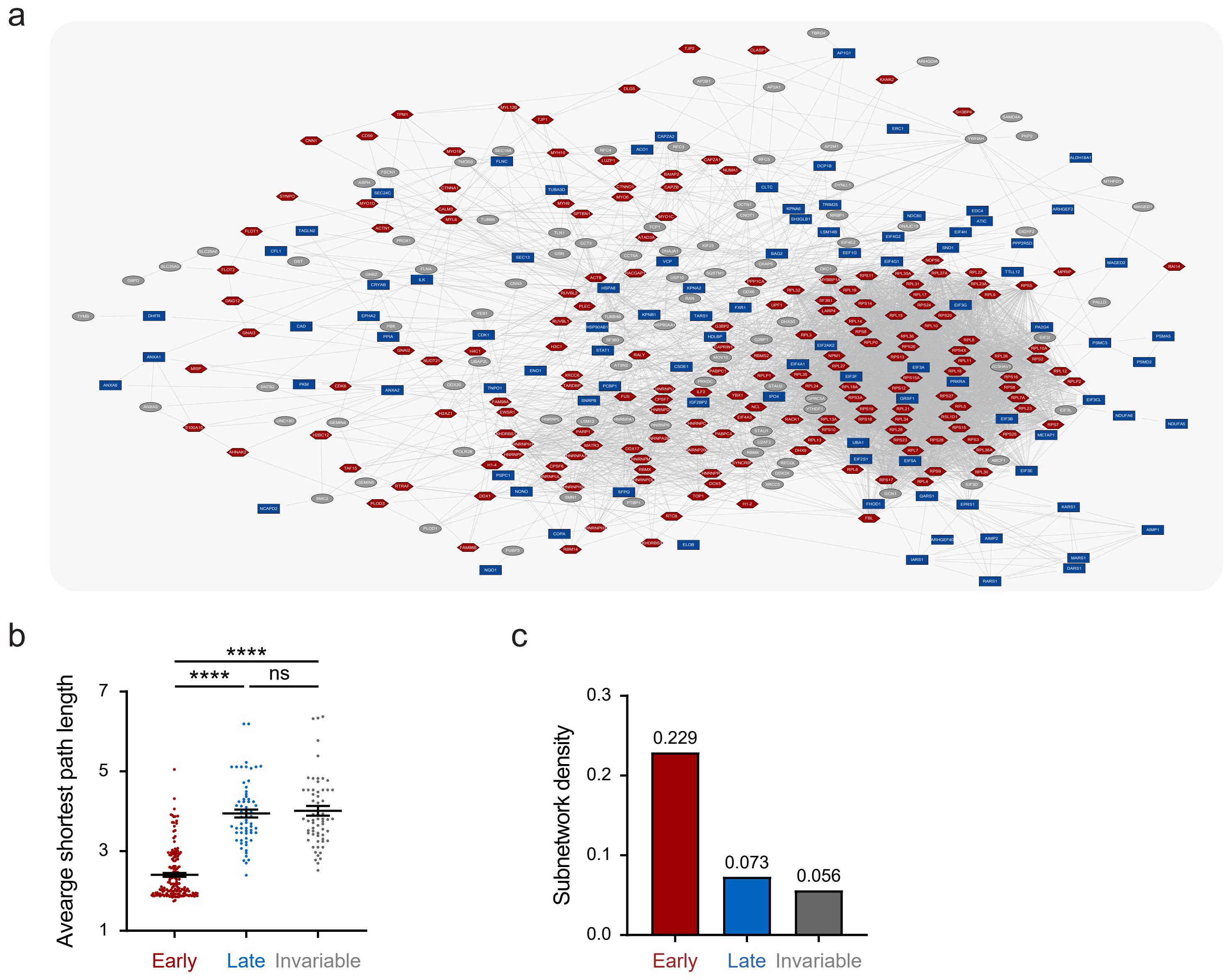

Supplementary Fig. 3. The SG PPI network, and comparison of topological properties among the three SG protein groups, related to Figure 3.

a. The SG PPI network with one connected component; nodes of early dynamic SG proteins, late dynamic SG proteins, and invariable proteins are represented by red hexagons, blue rectangles, and grey ovals, respectively.

b. Scatter plot showing the average shortest path length for subnetworks of the early dynamic (red), late dynamic (blue), and invariable (grey) proteins; n = 166, 64, and 63 proteins for early, late and invariable proteins, respectively; error bars indicate SEM; ****P < 0.0001, ns not significant (One-Way ANOVA with Tukey’s test, exact P values: early vs. late, early vs. invariable, < 0.0001; late vs. invariable, 0.8683).

c. Bar graph showing the network density for subnetworks of the early dynamic (red), late dynamic(blue), and invariable (grey) SG proteins.

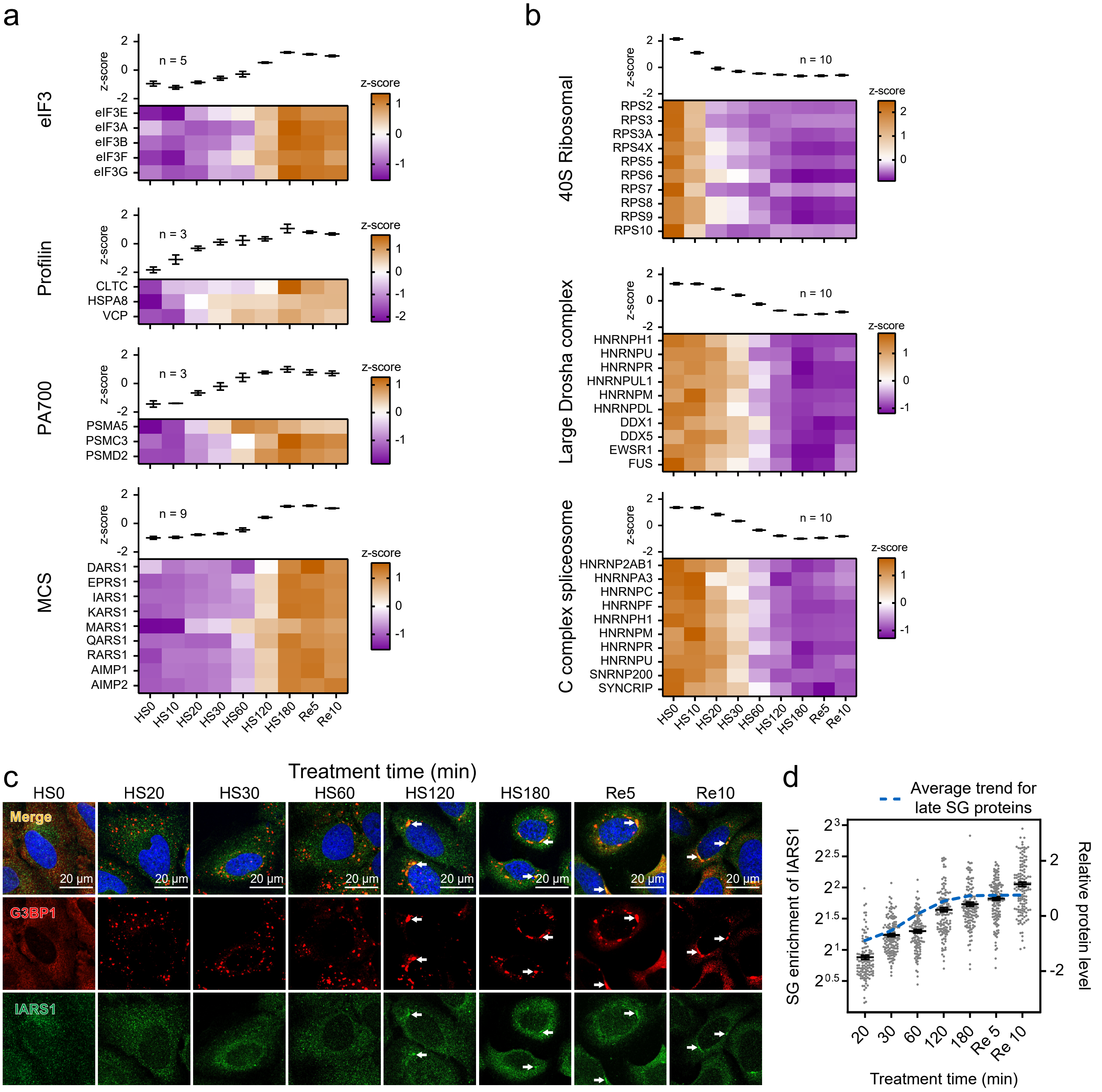

Supplementary Fig. 4. SG-enrichment profile of different protein complexes across the HS and recovery cycle, related to Figures 4, 5.

a. SG profiles for complexes enriched in late dynamic SG protein; bottom of each panel, heatmap showing changes of relative protein levels across the HS and recovery cycle; top of each panel, the average trend of all detected subunits in the complex; error bars indicate SEM.
b. SG profiles for complexes enriched in early dynamic SG proteins; bottom of each panel, heatmap showing changes of relative protein levels across the HS and recovery cycle, 10 proteins of each complex are shown; top of each panel, the average trend of all detected subunits in the complex; error bars indicate SEM.
c. Confocal micrographs of U2OS cells fixed at the indicated time points during HS and recovery; immunofluorescence of G3BP1 and IARS1 are shown in red and green, respectively; scale bars, 20μm; arrows highlight colocalization of two proteins; representative images from 3 biological replicates are shown.
d. Enrichment of IARS1 fluorescence intensity in G3BP1-positive granules over that in the whole cell, calculated per cell; error bars indicate SEM; the average SG-enrichment trend of late SG proteins based on proteomic profiling results is shown in blue as a reference; n = 123 cells for every time point, from 3 biological replicates.

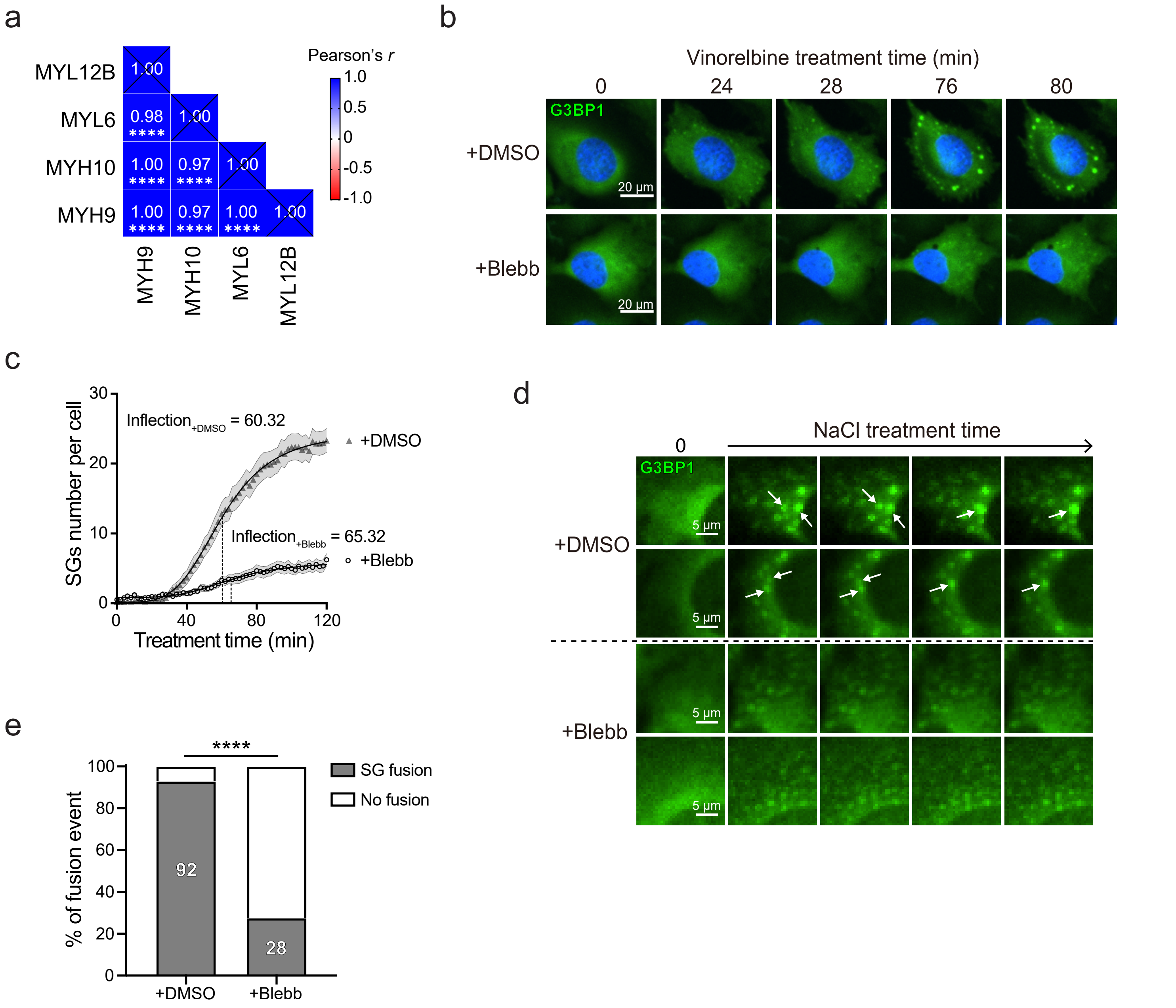

Supplementary Fig. 5. The NM II complex promotes SG formation, related to Figure 6.

a. A correlation matrix for SG profiles of all four subunits in the NM II complex; Pearson's r values are indicated by color and labeled; ****P < 0.0001 (Pearson's r were determined through pairwise comparisons, n = 24, including 3 replicates of 8 time points (HS10 to Re10) for each protein. P values were calculated using two-sided t-test).
b. Snapshots from live cell imaging of U2OS cells stably expressing EGFP-G3BP1, pre-treated with either DMSO or para-nitro-blebbistatin; imaging started at the induction of stress with vinorelbine; scale bars, 20 μ m; representative images from 3 biological replicates are shown.
c. Quantification of SG number per cell at different stress time; based on live cell imaging of U2OS cells stably expressing EGFP-G3BP1, pre-treated with either DMSO or para-nitro-blebbistatin, and stressed with vinorelbine; n = 51 (DMSO) or 46 (para-nitro-blebbistatin) cells from 3 biological replicates; shadows indicate SEM; estimated inflection points for fitted curves are labeled on the graph and shown as dash lines.
d. Snapshots from live cell imaging of U2OS cells stably expressing EGFP-G3BP1, pre-treated with either DMSO or para-nitro-blebbistatin; imaging started at the induction of osmotic stress with NaCl; scale bars, 5 μ m; Arrows indicate the fusion of G3BP1-positive granules; representative images from 3 biological replicates are shown.
e. Bar graph showing the percentage of manually examined ROIs with SG fusion event; quantification based on live cell imaging of osmotic stressed U2OS cells pre-treated with either DMSO or para-nitro-blebbistatin; n = 50 cells from 3 biological replicates; ****P < 0.0001 (two-sided Chi-square test).
