# Supplementary material for: Time-resolved proteomic profiling reveals compositional and functional transitions across the stress granule life cycle
Source: Nat Commun. 2023 Nov 27;14:7782. doi: 10.1038/s41467-023-43470-1 (PMC10682001; doi:10.1038/s41467-023-43470-1)
Supplement: Supplementary file 3 — Description of Additional Supplementary Files [file 41467_2023_43470_MOESM3_ESM.pdf]

Description of Additional Supplementary Files

Legends of Supplementary Data:

File Name: Supplementary Data 1

Description: Stress granule proteomic profiling data, including proteins list and previously reported SG constituent proteins list, related to Figure 1.

File Name: Supplementary Data 2

Description: SG-ls enrichment scores, related to Figure 2.

File Name: Supplementary Data 3

Description: GO Process enrichment results and CORUM enrichment results, related to Figure 3d and Supplementary Figure 4a-b.

Legends of Supplementary Movies:

File Name: Supplementary Movie 1

Description: Osmotic-stress-induced SGs in blebb-treated and vehicle-treated cells, related to Figure 6C.

File Name: Supplementary Movie 2

Description: Osmotic-stress-induced SGs in cells overexpressed either MYL9 or MYL9T18D/S19D, related to Figure 6G.
