# Supplementary material for: Time-resolved proteomic profiling reveals compositional and functional transitions across the stress granule life cycle
Source: Nat Commun. 2023 Nov 27;14:7782. doi: 10.1038/s41467-023-43470-1 (PMC10682001; doi:10.1038/s41467-023-43470-1)
Supplement: Supplementary file 9 — Reporting Summary [file 41467_2023_43470_MOESM9_ESM.pdf]

nature portfolio

Corresponding author(s): Shuyao Hu
Last updated by author(s): Oct 24, 2023

Reporting Summary

Nature Portfolio wishes to improve the reproducibility of the work that we publish. This form provides structure for consistency and transparency in reporting. For further information on Nature Portfolio policies, see our Editorial Policies and the Editorial Policy Checklist.

Statistics

For all statistical analyses, confirm that the following items are present in the figure legend, table legend, main text, or Methods section.

n/a	Confirmed	
☐	☒	The exact sample size (*n*) for each experimental group/condition, given as a discrete number and unit of measurement
☐	☒	A statement on whether measurements were taken from distinct samples or whether the same sample was measured repeatedly
☐	☒	The statistical test(s) used AND whether they are one- or two-sided<br>*Only common tests should be described solely by name; describe more complex techniques in the Methods section.*
☒	☐	A description of all covariates tested
☒	☐	A description of any assumptions or corrections, such as tests of normality and adjustment for multiple comparisons
☐	☒	A full description of the statistical parameters including central tendency (e.g. means) or other basic estimates (e.g. regression coefficient) AND variation (e.g. standard deviation) or associated estimates of uncertainty (e.g. confidence intervals)
☐	☒	For null hypothesis testing, the test statistic (e.g. *F*, *t*, *r*) with confidence intervals, effect sizes, degrees of freedom and *P* value noted<br>*Give P values as exact values whenever suitable.*
☒	☐	For Bayesian analysis, information on the choice of priors and Markov chain Monte Carlo settings
☐	☒	For hierarchical and complex designs, identification of the appropriate level for tests and full reporting of outcomes
☐	☒	Estimates of effect sizes (e.g. Cohen's *d*, Pearson's *r*), indicating how they were calculated

Our web collection on statistics for biologists contains articles on many of the points above.

Software and code

Policy information about availability of computer code

Data collection	Proteomic data were collected on an EASY-nLC 1200 system coupled to a Q Exactive HF (Thermo Scientific).<br>Immunofluorescence images were collected using Leica SP8 STED 3X inverted microscope with LAS X (Leica, 3.5.7.23225) software.<br>Live cell imaging was acquired using Nikon Ti2-E inverted microscope equipped with CSU-W1 spinning disk confocal scanner (Yokogawa) and live cell maintaining system (oko lab), with NIS-Elements AR (Nikon, 5.20.00) software.<br>Immunobloting bands were visualized using Amersham Imager 680 with IQ800 Control software (GE Healthcare, 1.2.0).
Data analysis	The raw proteomic data were searched using Proteome Discoverer (Thermo Scientific, 2.2).<br>The search results were subjected to the ComBat method from the sva R package (3.44.0; R version: 4.2.1).<br>Cluster analysis for the dynamic proteins were performed using ClustVis 2.0 (https://biit.cs.ut.ee/clustvis/).<br>The previously reported SG constituents list was downloaded from the RNA Granule Database 2.0 (https://rnagranuledb.lunenfeld.ca/).<br>Protein sequences were retrieved from the UniProt database (https://www.uniprot.org/).<br>Sequence based-IDR prediction was performed using IUPred3 (https://iupred.elte.hu/).<br>Sequence-based predication for PrLDs was performed using PLAAC (http://plaac.wi.mit.edu/).<br>PPI network were constructed using STRING 12.0 (https://string-db.org/) and Bioplex 3.0 (https://bioplex.hms.harvard.edu/), visualized using Cytoscape (3.9.1).<br>Topological features of the networkes were calculated using NetworkAnalyzer (4.4.8) in Cytoscape and NAP(v2.0): The Network Analysis Profiler (https://bib.fleming.gr:8084/app/nap).<br>GO-enrichment analysis was performed using Metascape 3.5.20230501 (http://metascape.org/gp/index.html#/main/step1).<br>Images of immunofluorescence and live cell imaging were analyzed and processed using Fiji ImageJ (1.53t).<br>Particle analysis was performed using the Particle Tracker 2D/3D feature of the Mosaic Plugin for ImageJ.

The statistical analysis and present of figures were processed using GraphPad Prism (9.0) or Excel (16.0.16827.20166).
The figure organization was using Adobe Illustrator (25.0).

For manuscripts utilizing custom algorithms or software that are central to the research but not yet described in published literature, software must be made available to editors and reviewers. We strongly encourage code deposition in a community repository (e.g. GitHub). See the Nature Portfolio guidelines for submitting code & software for further information.

Data

Policy information about availability of data

All manuscripts must include a data availability statement. This statement should provide the following information, where applicable:

- Accession codes, unique identifiers, or web links for publicly available datasets
- A description of any restrictions on data availability
- For clinical datasets or third party data, please ensure that the statement adheres to our policy

The RNA granule database is available at https://rnagranuledb.lunenfeld.ca/.
The mass spectrometry data has been deposited in the ProteomeXchange member repository, MassIVE, under the accession code PXD044967 [https://massive.ucsd.edu/ProteoSAFe/QueryPXD?id=PXD044967].
The source data generated in this study are provided in the Source Data file.
Further information and requests for resources and reagents should be directed to and will be fulfilled by the lead contact, Shuyao Hu (hushy@shanghaitech.edu.cn).
Requests of cell lines described in this study will be available upon request from the lead contact, under a standard MTA.

Research involving human participants, their data, or biological material

Policy information about studies with human participants or human data. See also policy information about sex, gender (identity/presentation), and sexual orientation and race, ethnicity and racism.

Reporting on sex and gender	N/A
Reporting on race, ethnicity, or other socially relevant groupings	N/A
Population characteristics	N/A
Recruitment	N/A
Ethics oversight	N/A

Note that full information on the approval of the study protocol must also be provided in the manuscript.

Field-specific reporting

Please select the one below that is the best fit for your research. If you are not sure, read the appropriate sections before making your selection.

☒ Life sciences ☐ Behavioural & social sciences ☐ Ecological, evolutionary & environmental sciences

For a reference copy of the document with all sections, see nature.com/documents/nr-reporting-summary-flat.pdf

Life sciences study design

All studies must disclose on these points even when the disclosure is negative.

Sample size	Three biological independent replicates were used for every experiment.<br>At least 100 cells were chosen to determine statistically significance in immunofluorescence experiments.<br>In live cell imaging, at least 30 cells were selected to ensure statistical significance because the number of cells captured in a single experiment was lower than that on fixed cell slides.
Data exclusions	No data were excluded from the analyses.
Replication	Three biological replicates were performed for each experiment.<br>All attempts at replication were successful.
Randomization	This is not relevant to our study.
Blinding	For the quantification of SG fusion events, images from both conditions were mixed before manually analyzed independently by two different researchers, they were blinded to group allocation during data collection and/or analysis.<br>For all other experiments, blinding is not relevant.

Reporting for specific materials, systems and methods

We require information from authors about some types of materials, experimental systems and methods used in many studies. Here, indicate whether each material, system or method listed is relevant to your study. If you are not sure if a list item applies to your research, read the appropriate section before selecting a response.

Materials & experimental systems

n/a	Involved in the study
☐	☒ Antibodies
☐	☒ Eukaryotic cell lines
☒	☐ Palaeontology and archaeology
☒	☐ Animals and other organisms
☒	☐ Clinical data
☒	☐ Dual use research of concern
☒	☐ Plants

Methods

n/a	Involved in the study
☒	☐ ChIP-seq
☒	☐ Flow cytometry
☒	☐ MRI-based neuroimaging

Antibodies

Antibodies used

Antibodies used in immunofluorecense:
mouse anti-G3BP1 monoclonal [2F3] antibody (Abcam, ab56574, 1: 500);
rabbit anti-G3BP1 polyclonal antibody (Proteintech, 13057-2-AP, 1: 500);
rabbit anti-CAPRIN1 polyclonal antibody (Proteintech, 15112-1-AP, 1: 500);
rabbit anti-PARP polyclonal antibody (Cell Signaling, 9542S, 1: 100);
rabbit anti-VCP monoclonal [EPR3307(2)] antibody (Abcam, ab109240, 1: 100);
rabbit anti-SEC24C polyclonal antibody (Abcam, ab122633, 1: 100);
rabbit anti-eIF3A polyclonal antibody (Cell Signaling, 2538S, 1: 50);
rabbit anti-S6 ribosomal protein monoclonal (5G10) antibody (Cell Signaling, 2217S, 1: 200);
rabbit anti-TDP-43 polyclonal antibody (Proteintech, 10782-2-AP, 1: 200), is a gift from Zhi Zhou's Lab, SLST, ShanghaiTech University;
rabbit anti-IARS1 polyclonal antibody (Proteintech, 26942-1-AP, 1: 50);
Donkey anti-Rabbit IgG (H+L) Secondary Antibody, Alexa Fluor 488 (Invitrogen, A21206, 1: 500);
Donkey anti-Mouse IgG (H+L) Secondary Antibody, Alexa Fluor 568 (Invitrogen, A10037, 1: 500);
Antibodies used in immunoblotting:
rabbit anti-G3BP1 polyclonal antibody (Proteintech, 13057-2-AP, 1: 5000);
rabbit anti-eIF3A polyclonal antibody (Cell Signaling, 2538S, 1: 1000);
rabbit anti-DDX3 polyclonal antibody (Invitrogen, A300-474A, 1: 2000);
mouse anti LC3A monoclonal (166AT1234) antibody (Abcepta, AM1800A, 1: 1000);
mouse anti-LAMP1 monoclonal (H4A3) antibody (Santa Cruz, sc-20011, 1: 500);
rabbit anti-IARS polyclonal antibody (ABclonal, A10190, 1: 2000);
rabbit anti-RARS polyclonal antibody (ABclonal, A6307, 1: 2000);
rabbit anti-EPRS polyclonal antibody (ABclonal, A15245, 1: 2000);
rabbit anti-KARS monoclonal (ARC1765) antibody (ABclonal, A8648, 1: 2000);
rabbit anti-QARS polyclonal antibody (ABclonal, A6960, 1: 2000);
rabbit anti-β-Actin polyclonal antibody (Cell Signaling, 4967S, 1: 1000);
mouse anti-alpha-tubulin monoclonal (1E4C11) antibody (Proteintech, 66031-1-Ig, 1: 20000);
Peroxidase-AffiniPure Goat Anti-Rabbit IgG (H+L) (Jackson, 111-035-144, 1: 10000);
Peroxidase-AffiniPure Goat Anti-Mouse IgG (H+L) (Jackson, 115-035-146, 1: 10000).

Validation

mouse anti-G3BP1 monoclonal [2F3] antibody (Abcam, ab56574): validated by manufacturer [https://www.abcam.cn/products/primary-antibodies/g3bp-antibody-2f3-ab56574.html];
rabbit anti-G3BP1 polyclonal antibody (Proteintech, 13057-2-AP): validated by manufacturer [https://www.ptgcn.com/products/G3BP1-Antibody-13057-2-AP.htm]
rabbit anti-CAPRIN1 polyclonal antibody (Proteintech, 15112-1-AP): validated by manufacturer [https://www.ptgcn.com/products/CAPRIN1-Antibody-15112-1-AP.htm]
rabbit anti-PARP polyclonal antibody (Cell Signaling, 9542S): validated by manufacturer [https://www.cellsignal.com/products/primary-antibodies/parp-antibody/9542];
rabbit anti-VCP monoclonal [EPR3307(2)] antibody (Abcam, ab109240): validated by manufacturer [https://www.abcam.cn/vcp-antibody-epr33072-ab109240.html];
rabbit anti-SEC24C polyclonal antibody (Abcam, ab122633): validated by manufacturer [https://www.abcam.cn/sec24c-antibody-ab122633.html];
rabbit anti-eIF3A polyclonal antibody (Cell Signaling, 2538S): validated by manufacturer [https://www.cellsignal.com/products/primary-antibodies/eif3a-antibody/2538];
rabbit anti-S6 ribosomal protein monoclonal (5G10) antibody (Cell Signaling, 2217S): validated by manufacturer [https://www.cellsignal.com/products/primary-antibodies/s6-ribosomal-protein-5g10-rabbit-mab/2217];
rabbit anti-TDP-43 polyclonal antibody (Proteintech, 10782-2-AP): validated by manufacturer [https://www.ptglab.com/products/TARDBP-Antibody-10782-2-AP.htm];
rabbit anti-IARS1 polyclonal antibody (Proteintech, 26942-1-AP): validated by manufacturer [https://www.ptglab.com/products/IARS-Antibody-26942-1-AP.htm];
Donkey anti-Rabbit IgG (H+L) Secondary Antibody, Alexa Fluor 488 (Invitrogen, A21206): validated by manufacturer [https://

www.thermofisher.com/antibody/product/Donkey-anti-Rabbit-IgG-H-L-Highly-Cross-Adsorbed-Secondary-Antibody-Polyclonal/A-21206];
Donkey anti-Mouse IgG (H+L) Secondary Antibody, Alexa Fluor 568 (Invitrogen, A10037): validated by manufacturer [https://www.thermofisher.com/antibody/product/Donkey-anti-Mouse-IgG-H-L-Highly-Cross-Adsorbed-Secondary-Antibody-Polyclonal/A10037];
rabbit anti-DDX3 polyclonal antibody (Invitrogen, A300-474A): validated by manufacturer [https://www.thermofisher.com/antibody/product/DDX3-Antibody-Polyclonal/A300-474A];
mouse anti LC3A monoclonal (166AT1234) antibody (Abcepta, AM1800A): validated by manufacturer [https://www.abcepta.com.cn/products/AM1800a-LC3-Antibody-APG8];
mouse anti-LAMP1 monoclonal (H4A3) antibody (Santa Cruz, sc-20011): validated by manufacturer [https://www.scbt.com/p/lamp-1-antibody-h4a3];
rabbit anti-IARS polyclonal antibody (ABclonal, A10190): validated by manufacturer [https://abclonal.com.cn/catalog/A10190];
rabbit anti-RARS polyclonal antibody (ABclonal, A6307): validated by manufacturer [https://abclonal.com.cn/catalog/A6307];
rabbit anti-EPRS polyclonal antibody (ABclonal, A15245): validated by manufacturer [https://abclonal.com.cn/catalog/A15245];
rabbit anti-KARS monoclonal (ARC1765) antibody (ABclonal, A8648): validated by manufacturer [https://abclonal.com.cn/catalog/A8648];
rabbit anti-QARS polyclonal antibody (ABclonal, A6960): validated by manufacturer [https://abclonal.com.cn/catalog/A6960];
rabbit anti-β-Actin polyclonal antibody (Cell Signaling, 4967S): validated by manufacturer [https://www.cellsignal.com/products/primary-antibodies/b-actin-antibody/4967];
mouse anti-alpha-tubulin monoclonal (1E4C11) antibody (Proteintech, 66031-1-Ig): validated by manufacturer [https://www.ptgcn.com/products/tubulin-Alpha-Antibody-66031-1-Ig.htm];
Peroxidase-AffiniPure Goat Anti-Rabbit IgG (H+L) (Jackson, 111-035-144): validated by manufacturer [https://www.jacksonimmuno.com/catalog/products/111-035-144];
Peroxidase-AffiniPure Goat Anti-Mouse IgG (H+L) (Jackson, 115-035-146): validated by manufacturer [https://www.jacksonimmuno.com/catalog/products/115-035-146].

Eukaryotic cell lines

Policy information about cell lines and Sex and Gender in Research

Cell line source(s)	U2OS cells were from www.cellbank.org.cn (National Collection of Authenticated Cell Cultures)
Authentication	None of the cell lines were authenticated after purchase.
Mycoplasma contamination	Cell lines were tested negative for mycoplasma contamination.
Commonly misidentified lines (See ICLAC register)	No commonly misidentified lines were used

Plants

Seed stocks	*Report on the source of all seed stocks or other plant material used. If applicable, state the seed stock centre and catalogue number. If plant specimens were collected from the field, describe the collection location, date and sampling procedures.*
Novel plant genotypes	*Describe the methods by which all novel plant genotypes were produced. This includes those generated by transgenic approaches, gene editing, chemical/radiation-based mutagenesis and hybridization. For transgenic lines, describe the transformation method, the number of independent lines analyzed and the generation upon which experiments were performed. For gene-edited lines, describe the editor used, the endogenous sequence targeted for editing, the targeting guide RNA sequence (if applicable) and how the editor was applied.*
Authentication	*Describe any authentication procedures for each seed stock used or novel genotype generated. Describe any experiments used to assess the effect of a mutation and, where applicable, how potential secondary effects (e.g. second site T-DNA insertions, mosiacism, off-target gene editing) were examined.*
